# Investigating Curcumin/Intestinal Epithelium Interaction in a Millifluidic Bioreactor

**DOI:** 10.3390/bioengineering7030100

**Published:** 2020-08-26

**Authors:** Joana Costa, Vanessa Almonti, Ludovica Cacopardo, Daniele Poli, Simona Rapposelli, Arti Ahluwalia

**Affiliations:** 1 Research Center “E. Piaggio”, University of Pisa, 56122 Pisa, Italy; ludovica.cacopardo@ing.unipi.it (L.C.); danielepoli.ge@gmail.com (D.P.); arti.ahluwalia@unipi.it (A.A.); 2 LARF-DIMES, Department of Experimental Medicine, University of Genoa, 16126 Genoa, Italy; vanessaalmonti@gmail.com; 3 Centro 3R (Inter-University Center for the Promotion of the 3Rs Principles in Teaching & Research), 56122 Pisa, Italy; simona.rapposelli@unipi.it; 4 Department of Pharmacy, University of Pisa, 56126 Pisa, Italy

**Keywords:** curcumin, Caco-2 cells, fluidic systems, P-gp modulation, bioreactors, intestinal in-vitro models

## Abstract

Multidrug resistance is still an obstacle for chemotherapeutic treatments. One of the proteins involved in this phenomenon is the P-glycoprotein, P-gp, which is known to be responsible for the efflux of therapeutic substances from the cell cytoplasm. To date, the identification of a drug that can efficiently inhibit P-gp activity remains a challenge, nevertheless some studies have identified natural compounds suitable for that purpose. Amongst them, curcumin has shown an inhibitory effect on the protein in in vitro studies using Caco-2 cells. To understand if flow can modulate the influence of curcumin on the protein’s activity, we studied the uptake of a P-gp substrate under static and dynamic conditions. Caco-2 cells were cultured in bioreactors and in Transwells and the basolateral transport of rhodamine-123 was assessed in the two systems as a function of the P-gp activity. Experiments were performed with and without pre-treatment of the cells with an extract of curcumin or an arylmethyloxy-phenyl derivative to evaluate the inhibitory effect of the natural substance with respect to a synthetic compound. The results indicated that the P-gp activity of the cells cultured in the bioreactors was intrinsically lower, and that the effect of both natural and synthetic inhibitors was up modulated by the presence of flow. Our study underlies the fact that the use of more sophisticated and physiologically relevant in vitro models can bring new insights on the therapeutic effects of natural substances such as curcumin.

## 1. Background

Chemotherapy remains the preferred treatment in the case of advanced and/or metastatic neoplasms. Unfavorably, cancer cells have shown the ability to develop resistance to many different antineoplastic drugs, displaying an adaptation commonly mentioned as “pleiotropic resistance” or “multidrug resistance” (MDR) [1]. Cellular mechanisms linked to drug resistance can occur simultaneously or sequentially to pharmacological resistance in cells exhibiting a drug-resistant phenotype, which can be manifested through the activation of numerous proteins [2]. One of the proteins involved in MDR is the membrane glycoprotein (P-gp), which belongs to the class of plasma membrane efflux proteins. P-gp acts like a pump, extracting neutral or weak basic amphipathic substances, such as chemotherapeutic agents, from the cell cytoplasm. In fact, P-gp has been the main pharmacological target in combating MDR for several years [3].

In the human gastrointestinal tract, P-gp is found in high concentrations on the apical surfaces of the superficial columnar epithelial cells in the colon and distal small bowel. Its expression appears to increase when the tissues pass into the tumor state, as occurs in the colorectal epithelium [4]. Many attempts have been made to identify effective P-gp inhibitors, known as chemosensitizers or MDR modulators, which sensitize resistant cells to the action of cytotoxic drugs. The approaches used up to now have not returned significant results and have failed in clinical trials [1]. The first attempts to modulate P-gp were carried out by testing calcium-channel blockers and antisteroids (first-generation drugs), however these compounds interfered with several enzyme systems [5]. Later, second- and third-generation drugs were developed, but have failed in clinical trials mainly due to unpredictable pharmacokinetic interactions [6].

Alternatively, researchers have tried to find a valid P-gp inhibitor from substances of natural origins. Among the compounds studied, we can cite those deriving from black pepper (piperine, *Piper nigrum*) [7], sage (thanshinones, *Salvia miltiorrhiza*) [8], licorice (glycyrrhetinic acid, *Glycyrrhiza glabra*), chili pepper (capsaicin, *Capsicum annuum*) green tea ((-)-epigallocatechin gallate, *Camellia sinensis*) [9] and turmeric (curcumin, *Curcuma Longa*) [10].

**Figure 1:**
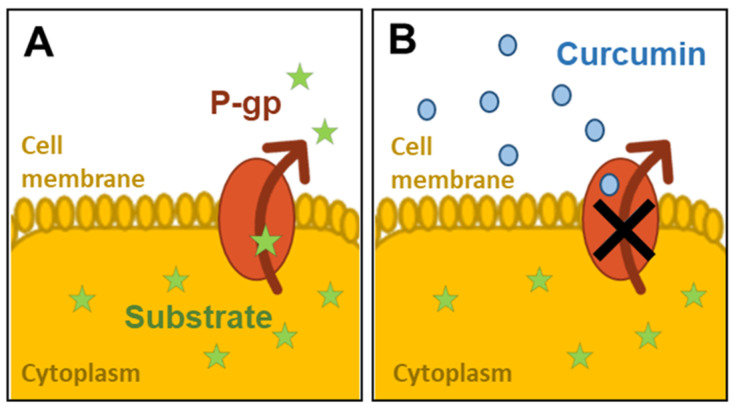
Inhibition of P-glycoprotein (P-gp) activity by curcumin: (**A**) the substrate is pumped out of the cell cytoplasm by P-gp; (**B**) the activity of P-gp is inhibited by the presence of curcumin molecules on the apical surface.

*Curcuma Longa* has been one of the most intensively studied plant extracts and its action on intestinal glycoproteins has been investigated in static in vitro models. Figure 1 illustrates the inhibition of the P-gp pumping activity in the presence of curcumin.

In a study using intestinal cell monolayers as a model of the gastrointestinal tract, the authors found that curcumin downregulated the expression and function of intestinal P-gp [11]. Specifically, curcumin at a concentration of 30 µM increased rhodamine 123 (Rh 123) (a substrate of P-gp) accumulation by two fold compared to the negative control and reduced efflux (by 30%) in the apical site. Ampasavate et al. investigated the P-gp modulating effects of extracts prepared from the rhizomes of Curcuma Longa and Curcuma sp. “Khamin oi”. They showed that the extract of Curcuma Longa increased Rh 123 uptake in a dose dependent manner, inhibiting the activity of intestinal P-gp. [10].

Both studies were performed using Caco-2 cells, cultured in a Transwell^®^ (Corning, New York, NY, USA) system, which differentiate to form a polarized epithelial cell monolayer providing a physical and biochemical barrier to the passage of ions and small molecules [12,13]. This cell line is widely used across the pharmaceutical industry as an in vitro model of the human small intestinal mucosa to predict the absorption of orally administered drugs [14].

In order to understand how the presence of flow could affect the modulation of P-gp, we studied the inhibition of intestinal P-gp by culturing Caco-2 cells in bioreactors. The systems we used combine a two-compartment cell culture device with apical and basal media flow and have been described by Giusti [15] and Cacopardo [16]. Both studies demonstrate that they are capable of better mimicking physiological barriers as they recapitulate the dynamic environment of the intestine. Here we compared the apical efflux of a P-gp substrate after preconditioning it with a curcumin extract and a synthetic compound (an arylmethyloxy-phenyl derivative or APD [17]) which proved to inhibit P-gp activity in a [^3^H]-vinblastine transport inhibition test with an IC_50_ of 0.19 µM. The experiments were performed in Transwells (traditional static system) and used bioreactors. To the best of our knowledge, this is the first application of such devices to the investigation of P-gp inhibition by curcumin.

## 2. Methods

**Figure 2:**
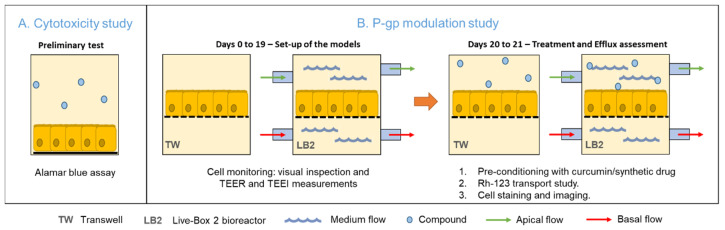
Representation of the experimental approach. (**A**) Preliminary study on the cytotoxicity of curcumin. (**B**) Set up of the static and dynamic in vitro models and P-gp activity assessment at the end of the 21-day culture period.

First, we performed a preliminary cytotoxicity test to determine a safe curcumin dose to be employed in the P-gp modulation study. Then, we set-up the culture of Caco-2 cells in the two systems for 21 days, with regular cell monitoring, including TEER and TEEI (trans epithelial electrical resistance and impedance, respectively). Finally, the P-gp activity in response to treatment with the two different compounds (curcumin extract and the synthetic compound APD) was assessed through a transport study using a P-gp substrate—rhodamine-123 (Rh-123). Figure 2 schematically illustrates the experiments performed. 

### 2.1. Cell Culture and Subculturing

Caco-2 cells from the American Type Culture Collection (ATCC) were grown in tissue culture flasks in Dulbecco’s Modified Eagle’s medium (high glucose) supplemented with 10% (*v*/*v*) heat-inactivated fetal bovine serum (FBS), 1% (*v*/*v*) L-glutamine, 1% (*v*/*v*) non-essential amino acids and 1% (*v*/*v*) penicillin/streptomycin. All reagents were purchased from Sigma-Aldrich, Italy, unless stated otherwise.

The LB2 bioreactors, purchased from IVTech srl, were composed of an apical and basal chamber, separated by a polyethylene terephthalate (PET) membrane (pore size: 0.45 µm; 2 × 10^6^ pores/cm^2^; ipCELLCULTURE, it4ip, Louvain-la-Neuve, Belgium). Both chambers are closed by two circular glass slides, one at the top and one at the bottom of the system, to allow live imaging. These are connected through fluidic tubing to separate the medium reservoirs (the mixing chambers). The flow was generated by a peristaltic pump (LiveFlow, IVTech Srl, Massarosa, Italy).

Caco-2 cells (between passages 30 and 45) were seeded at a density of 25,000 cells/cm^2^ in bioreactors (through their tubing system) and PET 12-well-plate Transwell^®^ (pore size: 0.40 µm; 4 × 10^6^ pores/cm^2^; Corning, New York, NY, USA). After cell adhesion, the apical chambers of the bioreactor were filled with 2 mL of medium and the basolateral ones with 1 mL of medium. In the Transwells, the apical and basolateral compartments were filled with 500 µL and 1 mL of medium, respectively. All the samples were incubated at 37 °C in a humidified atmosphere of 5% CO_2_ for three weeks. In the static samples the medium was changed every three days.

After one week, the basal and apical circuits were filled up to a total volume of 10 mL of media, respectively and kept in dynamic conditions (under laminar flow) at a flow rate of 150 µL/min, which according to the manufacturer should provide an average shear stress of around 6 × 10^−4^ Pa on the membrane, which is within the physiological range [18]. In this set-up, the medium was changed once a week: half the volume was removed and replaced by fresh medium. 

### 2.2. Curcuma Extract and APD Compound Preparation

The extract of turmeric was taken from a common encapsulated supplement (Curcumina Santé, Santé Naturels, Civitanova Marche, Italy) containing 450 mg of pure curcumin, titled at 95%. It is regularly registered as a food supplement notified to the Italian Ministry of Health. A stock solution of curcumin (45 mg/mL) was diluted in DMSO and stored at −20 °C.

APD (kindly provided by the Medicinal Chemistry Lab, Dept. of Pharmacy, UNIPI, Italy) was dissolved in Hank’s balanced salt solution (HBSS) and the stock solution (10 mM) was stored at −20 °C. This compound belongs to a new class of molecules that showed appreciable modulatory activity on P-gp and a high degree of selectivity [17,19,20]. The authors studied P-gp inhibition activity by 3 combined biological assays among which there was an inhibition in the P-gp mediated transport of vinblastine. This derivative was shown to compete with radiolabeled vinblastine (a well-known P-gp substrate) for the P-gp binding site, with an IC_50_ value of 0.19 µM. [17]. 

### 2.3. Assessment of Curcumin Toxicity 

The toxicity of curcumin was measured through a preliminary assessment of the metabolic activity of the cells, before and after incubation with the compound at different concentrations. This initial range-finding experiment was conducted to establish a curcumin dose which could be used in ensuing comparative tests between dynamic and static conditions.

Caco-2 cells were seeded in 24 well flat-bottom plates (Corning, New York, NY, USA) and cultured for 7 days. On the seventh day the medium was replaced with a fresh medium containing different concentrations of curcumin (11.25, 22.5, 45, 90 µg/mL), assuring the DMSO was present at a nontoxic concentration (0.02%). The cells were incubated with the different solutions at 37 °C for 48 h and the control samples solution contained only 0.02% DMSO in the cell medium.

### 2.4. Cell Barrier Monitoring and Morphological Analysis

The monolayer formation and maintenance were verified every three days with an optical microscope (Olympus AX81, Olympus, Segrate, Italy) both in the Transwell and in the bioreactors. Moreover, the transepithelial electric resistance (TEER) measurements, which are related to tight junction formations and thus provide an indication of the cellular layer tightness, were performed. 

Usually, TEER is measured applying a low-frequency (f < 5 kHz) current stimulus across the cellular barrier and recording the resulting voltage [16,21]. In this study, it was monitored at 40 Hz with an integrated cellular impedance meter [16,22] in the bioreactors at 12.5 Hz with an epithelial voltohmmeter (EVOM, World Precision Instruments, Sarasota, FL, USA) provided with chopstick electrodes in the Transwells. As reported by Cacopardo et al. [16], the measurements performed by the two instruments are equivalent.

The final TEER values were obtained by subtracting the blank resistance values related to the membrane and the culture medium, and multiplying them by the surface area of the respective membrane (i.e., 1.2 cm^2^ for the TW and 1.8 cm^2^ for the bioreactors). The impedance spectra were also acquired in the bioreactors within a frequency range of 40–10,000 Hz, providing a more precise indication regarding the formation of a tight monolayer. The TEEI values were obtained by subtracting the blank impedance values and multiplying them by the membrane area. 

### 2.5. Assessment of P-gp Activity 

On the twentieth day of culture, all the samples were rinsed with phosphate-buffered saline (PBS) and then incubated with a curcumin solution (50 µg/mL) that was added to the apical compartments through the mixing chambers in the bioreactors (10 mL) and directly in the apical compartment in the Transwells (0.5 mL). The basolateral compartments were filled with fresh medium—10 mL for the bioreactors and 1 mL for the Transwells. The flow rate was set to 150 µL/min in both circuits. All samples were incubated at 37 °C overnight.

After the treatment, the curcumin solution was removed, and the samples were washed with PBS. The activity of P-gp was then evaluated using a fluorescent compound rhodamine-123 (Rh-123) (Sigma-Aldrich, Milan, Italy), which is a tracer dye used as a substrate in the functional studies of MDR phenotype cells and, in particular, of the P-glycoprotein [23,24]. HBSS (ThermoFisher Scientific, Monza, Italy) was added in the apical compartments and the Rh-123 solution (10 µM in HBSS) in the basolateral ones. The same procedure was carried out for the Transwell samples. At the time-points of 0 and 120-min, 100 µL samples were collected from the apical sites and their volumes were replaced with pre-warmed HBSS. After two hours the samples were also collected from the basolateral ones. The Rh-123 concentration was determined using a plate reader (PerkinElmer, Hopkinton, MA, USA) setting filter wavelengths to 485 nm (excitation) and 535 nm (emission). 

Positive and negative control experiments were performed, repeating the protocol. In the positive one, APD (100 µM) was added to the apical sites of models instead of curcumin. The aim of this test was to verify the inhibition of P-gp activity by the third-generation drug, together with the lower passage of Rh-123 on the apical side of the cells.

**Figure 3:**
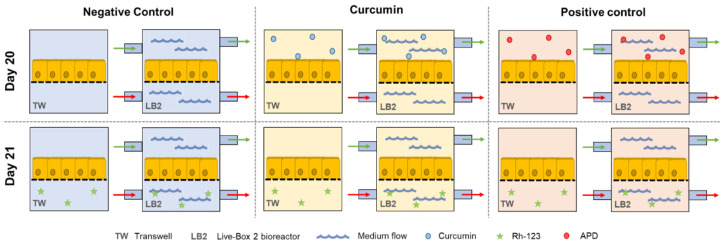
Schematic representation of the six different sample groups for the study of P-gp activity.

Finally, a negative control to measure the basal activity of P-gp in physiological conditions, without any cell preconditioning, was performed. All the experiments were conducted in parallel in the two in vitro models (dynamic and static) for each of the three conditions, with n = 6 experiments per condition. The three different experiments are illustrated in Figure 3.

Prior to the cell studies, the same transport experiments in both the Transwells and bioreactors were performed in the absence of cells. The residual amount of Rh-123 that remained bound to the material was considered and used to normalize the values obtained with the cells.

### 2.6. Data Analysis 

The analysis of the effect of curcumin or APD on P-gp activity is based on the Rh-123 efflux from the basolateral side to the apical side of planar cell cultures. Mathematically, P-gp activity is expressed as a function of the Rh-123 mass (µg) on both apical and basal sides at 120 min (t1_apical_ and t1_basal_, respectively), as well as Rh-123 initially administered to the experimental set-up (t0_basal_). All these parameters were normalized with respect to the blanks (i.e., measurements without cells) and combined into the equation as follows:

$$\mathrm{P-gp}\;activity\%=\frac{t1_{apical}}{t0_{basal}-t1_{basal}}\times 100$$

To appreciate how much the treatment with the substances (curcumin vs. APD) inhibited the activity of the protein, the following equation was used:

$$Inhibition\%=\frac{\mathrm{P-gp}\;activity{\;}_{negative\;control}-\mathrm{P-gp}\;activity_{substance}\;}{\mathrm{P-gp}\;activity_{negative\;control}}\times 100$$

Statistics based on a two-way ANOVA analysis was performed using the Tukey’s test for multiple comparisons and setting the statistical significance at *p* < 0.05, with GraphPad Prism (GraphPad Software, San Diego, CA, USA). The error bars in the results section represent the standard deviations (n = 6). 

## 3. Results 

### 3.1. Assessment of the Toxicity of Curcumin 

**Figure 4:**
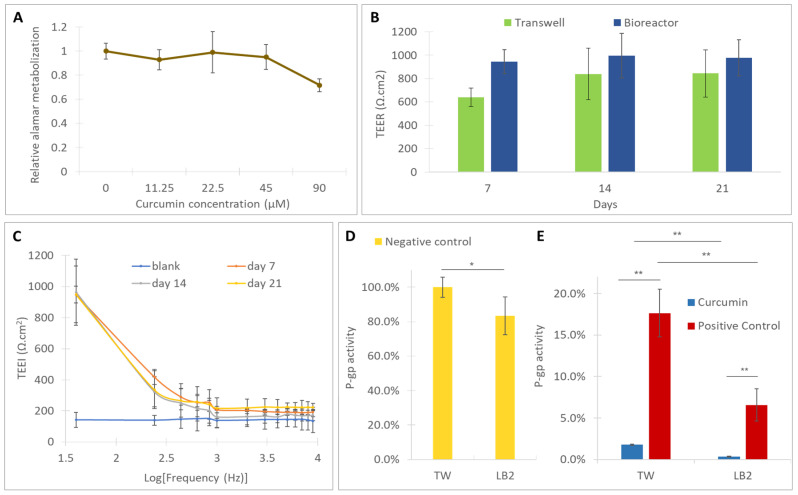
(**A**) Cell vitality, with respect to the 0 µM control, for different concentrations of curcumin after 48h of incubation; barrier monitoring through (**B**) trans epithelial electrical resistance (TEER) in the bioreactors and Transwells, (**C**) trans epithelial electrical impedance (TEEI) in the bioreactors at different timepoints; (**D**) P-gp activity in untreated samples (negative controls); (**E**) P-gp activity modulation by curcumin and arylmethyloxy-phenyl derivative (APD) (positive control) in Transwells and in the bioreactors. Error bars represent the standard deviations (n = 6). * = *p* < 0.05, ** = *p* < 0.01.

The cytotoxicity of curcumin on Caco-2 cells was assessed by incubating the cells with different concentrations of curcumin. The cells were grown in a 24-well-plate for 7 days and their metabolic activity was determined by performing the Alamar blue assay, before and after 48 h incubation with the different curcumin extracts. The results (Figure 4A) showed that the metabolic activity of the cells was not substantially affected by curcumin solutions in the concentration range 45–11.25 µM. Only for the highest concentration tested, 90 µM, did the cell metabolic activity decrease down to roughly 70%. A 50 µM curcumin solution was within the nontoxic range, so it was used for the P-gp activity modulation experiments.

### 3.2. Cell Monitoring and Morphological Analysis

After the seeding in the bioreactors and in the Transwells, the Caco-2 cells were regularly monitored over a 21 day differentiation period, using an optical microscope and by measuring the TEER and TEEI.

**Figure 5:**
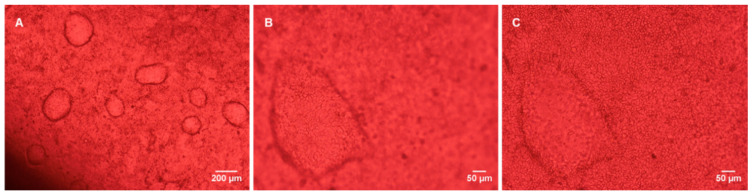
Caco-2 monolayer in the bioreactor at culture day 11. (**A**) 4× magnification; (**B**,**C**) 10× magnification (at different focal planes).

The cells progressively formed a compact monolayer, exhibiting features of differentiated cells after 10–12 days, as shown by the images in Figure 5.

The formation of a compact monolayer was also verified by the TEER and TEEI analysis. As shown in Figure 4B, the TEER increased during the culture reaching a plateau at day 14 (~800 Ω·cm^2^ in the Transwells and ~1000 Ω·cm^2^ in the bioreactors) and was maintained until day 21. Moreover, the TEEI trends (Figure 4C), measured in the bioreactors, suggest the presence of an integral cell layer at all the time points [16]. 

**Figure 6:**
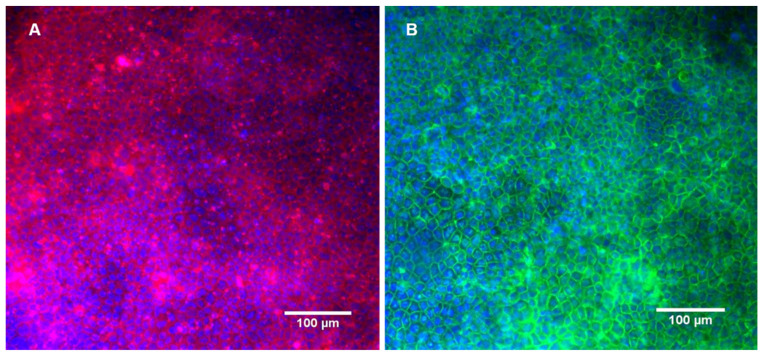
Caco-2 monolayer cultured in the bioreactor after the incubation with curcumin and subsequent P-gp activity assay. Fluorescence staining of the nuclei, actin microfilaments (**A**) and occludin (**B**).

After the P-gp modulation experiment, the cells were fixed and the actin fibers and the tight junction protein occludin were stained. The monolayer was remained intact during the transport assay and the cells preserved their tight junctions, as pointed out by the presence of occludin (Figure 6B).

### 3.3. Assessment of the Activity of P-gp

We measured P-gp activity as a function of the Rh-123 efflux from the basal side to the apical side of cells cultured in the Transwell and in the bioreactors. Additionally, we assessed the modulation of curcumin and APD (i.e., positive control) by their inhibition effect.

The statistical analysis showed that, in the absence of inhibitors, the P-gp activity observed in the bioreactors is lower than the activity measured in static conditions (Figure 4D). In the presence of natural and synthetic inhibitors, P-gp activity is further reduced in the bioreactors with respect to the Transwells (Figure 4E). Moreover, in both the static and dynamic conditions, the inhibition of P-gp activity by curcumin (average inhibition values of 98% in static conditions and 100% in dynamic conditions) is higher than that observed in the positive controls (average inhibition values of 83% in static conditions and 92% in dynamic conditions) demonstrating that the natural substance more markedly affects the Rh-123 efflux than the selective P-gp modulator APD. 

## 4. Discussion

In the preliminary cytotoxicity test, we observed a decrease in Caco-2 metabolic activity (<20%) at the maximum curcumin concentration tested (90 µM). Therefore, a nontoxic concentration of 50 µM curcumin was used for the P-gp modulation experiment. Similar concentrations have been employed in other reports in the literature [10,25,26].

Caco-2 cells grown in static Transwells and in bioreactors formed tight and well differentiated monolayers. It is worth noticing that the expansion of cells in the monolayer did not occur on a single plane, but gave rise to domes, as shown in Figure 5B,C, resembling the natural arrangement of cells in the intestinal environment [27]. Both in the Transwells and in the bioreactors, it was possible to observe the progressive formation of the monolayer, which, as illustrated in Figure 4B, came to confluence in about seven days (TEER values higher than 600 Ω·cm^2^) reaching a maximum at day 14 and 21 (~800 Ω·cm^2^ in the Transwells and ~1000 Ω·cm^2^ in the bioreactors). The integrity of the monolayer in the bioreactors was confirmed by the TEEI measurements (Figure 4C), which follow a typical RC circuit trend. Indeed, the cell monolayer can be represented by an equivalent electric circuit composed of the resistive paracellular pathway and cell capacity (transcellular pathway) in parallel [28,29]. At lower frequencies, the capacitor becomes progressively more charged and the current flow across it decreases, thus ions flow paracellulary (i.e., through the tight junctions). The higher the density and tightness of tight junctions, the greater the low frequency resistance. At a higher frequency, the capacitor becomes progressively more conductive and, in the presence of a compact cell layer with well-established tight junctions, the current is able to flow across the cells (transcellular current). Higher TEER values in the bioreactors, with respect to the Transwells, suggest that the fluidic conditions improve the barrier’s integrity and tightness, as reported by Cacopardo et al. (2019) [16].

The presence of tight junctions was also confirmed by occluding staining, after 21 days in culture and after the passage test. Occludin is a protein that protrudes on the outer face of the membrane and mediates cell to cell adhesion. It has also been linked with the regulation of intermembrane and the paracellular diffusion of small molecules [30]. Furthermore, as demonstrated in Figure 6, the P-gp activity assessment experiment did not interfere with the monolayer integrity and tightness. 

The effect of curcumin on P-gp was evaluated through the efflux of The rhodamine-123 from the basal side to the apical side of the cells. Since P-gp is a membrane glycoprotein, expressed especially on the apical side of cells, the presence of rhodamine-123 on the apical side can be correlated with its activity [4,31].

Our results indicated that curcumin strongly inhibited P-gp activity in the static model, which is in line with the previously described studies performed in the absence of flow [10,11]. Moreover, using bioreactors, media flow was applied to intestinal cells at the liquid–liquid interface in an attempt to better mimic the in vivo environment. The data show that the inhibition effect of curcumin is even more marked in dynamic conditions, and to our knowledge this aspect has not been considered in the investigation of the therapeutic effects of curcumin. Both the static and dynamic results reflect reports in mouse and rat models where curcumin was shown to affect the pharmacokinetics of orally administered and perfused anticancer drugs, through the reduction in the P-gp activity [32,33]. 

Regarding the inhibition by the synthetic modulator, the data collected to date on APD show that it inhibits the activity of P-gp at even lower doses than used in this study (100 µM) [17] Interestingly, we observed that the inhibition of P-gp by APD was surpassed by the inhibitory effect of curcumin (Figure 4E) in both static and dynamic conditions. As curcumin may also interact with other ATP-binding cassette (ABC)-transporters expressed in Caco-2 cells (such as MRP2 and BCRP/ABCG2 [34,35]) for which APD does not show any activity [17], further investigations should be performed to confirm the higher inhibitory action of curcumin, with respect to APD on P-gp activity. 

Regardless of the different potency of the two compounds towards P-gp, the main novelty of this study lies in the analysis of the effects of fluid flow on the apical and basolateral sides of the cells on the modulation of the P-gp protein activity. The results show that there are notable differences in the passage and pumping of Rh-123 between the cells cultured on Transwell membranes and the cells cultured in the bioreactors and, in all cases, P-gp’s efflux related activity was lower in dynamic conditions (Figure 4D,E). This highlights the advantage of using bioreactors for Caco-2 models, since the drug efflux mediated by the P-gp is often overestimated in the traditional monolayer model [36].

We propose that the P-gp’s efflux differences between static and dynamic conditions could be due to mechanotransduction effects arising from shear stress, which are known to modulate P-gp activity [37]. Other studies have assessed the effect of fluid flow on P-gp expression, but not on its modulation using natural compounds. Based on the apparent permeability values reported by Pusch et al., the basal–apical transport of rhodamine in a 2D static Caco-2 culture was higher than in a dynamic 3D tissue equivalent. The authors did not compare 2D static and 2D dynamic conditions [27]. 

On the other hand, Deng et al. [38] reported that Caco-2 cells cultured in a hollow fiber bioreactor revealed a higher expression of P-gp, which was attributed to the topographic features of the substrate. Reporting a similar result, Schweinlin et al. [39] observed that the expression of P-gp and efflux of Rh-123 increased in a decellularized biological scaffold-based multicellular model of the intestinal barrier cultured in a perfusion bioreactor, comparing with the values obtained for a Caco-2 monoculture from another study. 

It should be taken into account that the previously mentioned studies reported different bioreactors, substrates and/or cell sources, arrangements and compositions, which could justify the discrepancies in the events observed regarding P-gp activity, since the inclusion of different biochemical and mechanical cues can have potential synergistic effects [40]. 

It is also worth noticing that differing events regarding the modulation of the protein by the presence of fluid flow have not only been reported in intestinal models. For instance, a study by Garcia-Polite et al. demonstrated that the protein activity had a peak when endothelial cells were subjected to a shear stress of 1 Pa and that it decreased for a shear stress of 4 Pa [41]; while there is a contrasting hypothesis that P-gp is downregulated in the arterioles of rat brains in response to a higher shear stress [42,43]. Nevertheless, the shear stress provided by the flow in the present study is below the mentioned ranges and suggests that these cells are highly sensitive to even low levels of mechanical stimuli. It should however be noted that the analysis of the effects of an increased shear stress are very difficult to decouple from those due to an increased nutrient supply [44].

Remarkably, although the P-gp activity of the cells in the bioreactors was intrinsically lower, the activity inhibition efficiency, by both the ADP and curcumin, was higher in the bioreactors. This suggests that the presence of a flow not only influences P-gp by itself, but also affects the action of external modulators. These contrasting events could be due to the effect of flow on the binding between the substrate (Rh-123) and the protein and/or on the inhibition mechanism by both the arylmethyloxy-phenyl derivative and curcumin. 

It should be noted that the pharmacokinetic properties of curcumin in humans are not completely elucidated. It is known that, when ingested, the substance has a low oral bioavailability in humans [45] but it has been employed in several clinical trials of anticancer substances, with results reviewed in a paper by Kunnumakkara et al. [46]. The proposed dynamic model constitutes a more faithful representation of the microenvironment of the human intestinal epithelium in vitro, enabling a better translation between in vivo and in vitro studies. As the first such study on the modulation of P-gp by curcumin, it highlights the need to use more physiologically relevant cell culture systems for the study of natural substances. Future in vitro studies should be directed at identifying the precise mechanisms by which P-gp modulation by curcumin is different in dynamic systems, and to simulate the pharmacodynamic events curcumin undergoes before reaching and after translocating through the intestinal barrier.

## 5. Conclusions

We show that the presence of a physiological microenvironment can affect how P-gp is modulated by exogenous substances in vitro. Our results indicate that there is a difference in the inhibition of the protein by the action of curcumin in dynamic and static models. The potential therapeutic application of natural substances is of interest both in drug development and nutraceuticals, and it is crucial to perform in vitro studies using conditions which replicate the in vivo environment as faithfully as possible. As such, the present study is the first evidence that the use of more sophisticated tools can bring new insights on the therapeutic effects of natural substances such as curcumin.

## List of Abbreviations

ABC	ATP-binding cassette
ABCG2	ATP binding cassette sub-family G member 2
APD	Arylmethyloxy-phenyl derivative
ATCC	American Type Culture Collection
ATP	Adenosine triphosphate
BCRP	Breast cancer resistance protein
Caco-2	Colorectal Adenocarcinoma cell line
DMSO	Dimethyl sulfoxide
EVOM	Epithelial VoltOhmMeter
FBS	Fetal Bovine Serum
HBSS	Hank’s Balanced Salt Solution
LB2	LiveBox2 bioreactor
MDR	Multidrug Resistance
MRP2	Multidrug resistance-associated protein 2
P-gp	P-glycoprotein
PBS	Phosphate-buffered saline
PET	Polyethylene terephthalate
Rh-123	Rhodamine 123
TEEI	Transepithelial Electrical Impedance
TEER	Transepithelial Electrical Resistance
TW	Transwell
